# Clinical and virological characteristics of dengue in Surabaya, Indonesia

**DOI:** 10.1371/journal.pone.0178443

**Published:** 2017-06-02

**Authors:** Puspa Wardhani, Aryati Aryati, Benediktus Yohan, Hidayat Trimarsanto, Tri Y. Setianingsih, Dwiyanti Puspitasari, Muhammad Vitanata Arfijanto, Bramantono Bramantono, Suharto Suharto, R. Tedjo Sasmono

**Affiliations:** 1 Department of Clinical Pathology, School of Medicine, Universitas Airlangga, Surabaya, Indonesia; 2 Institute for Tropical Diseases, Universitas Airlangga, Surabaya, Indonesia; 3 Eijkman Institute for Molecular Biology, Jakarta, Indonesia; 4 Department of Pediatric, School of Medicine, Universitas Airlangga, Surabaya, Indonesia; 5 Department of Internal Medicine, School of Medicine, Universitas Airlangga, Surabaya, Indonesia; University of Minnesota, UNITED STATES

## Abstract

Dengue disease is still a major health problem in Indonesia. Surabaya, the second largest city in the country, is endemic for dengue. We report here on dengue disease in Surabaya, investigating the clinical manifestations, the distribution of dengue virus (DENV) serotypes, and the relationships between clinical manifestations and the genetic characteristics of DENV. A total of 148 patients suspected of having dengue were recruited during February-August 2012. One hundred one (68%) of them were children, and 47 (32%) were adults. Dengue fever (DF) and Dengue hemorrhagic fever (DHF) were equally manifested in all of the patients. We performed DENV serotyping on all of the samples using real-time RT-PCR. Of 148, 79 (53%) samples were detected as DENV positive, with DENV-1 as the predominant serotype (73%), followed by DENV-2 (8%), DENV-4 (8%), and DENV-3 (6%), while 5% were mixed infections. Based on the Envelope gene sequences, we performed phylogenetic analyses of 24 isolates to genotype the DENV circulating in Surabaya in 2012, and the analysis revealed that DENV-1 consisted of Genotypes I and IV, DENV-2 was of the Cosmopolitan genotype, the DENV-3 viruses were of Genotype I, and DENV-4 was detected as Genotype II. We correlated the infecting DENV serotypes with clinical manifestations and laboratory parameters; however, no significant correlations were found. Amino acid analysis of Envelope protein did not find any unique mutations related to disease severity.

## Introduction

Dengue is a self-limited, systemic viral infection caused by dengue virus (DENV), a member of the Flaviviridae family. Dengue poses a significant public health challenges, with a global burden of an estimated 390 million infections per year occur across approximately 128 countries, with the potential for further spread [1–3]. Four DENV serotypes (DENV-1, -2, -3, and -4) circulate in tropical and subtropical regions of the world and are transmitted by *Aedes* mosquitoes as the vector [4].

The clinical manifestations of dengue range from asymptomatic or a mild flu-like syndrome known as classic Dengue fever (DF), to a more severe form known as dengue hemorrhagic fever (DHF) and the potentially fatal dengue shock syndrome (DSS) [5,6]. DF generally characterized by acute febrile illness, often accompanied with severe headache, myalgia, arthralgia, rashes, leukopenia and thrombocytopenia. Unusual haemorrhage such as gastrointestinal bleeding, hypermenorrhoea and massive epistaxis sometimes occur [6]. In DHF, the signs and symptoms during the early febrile phase are similar to those in DF. The distinct feature of DHF is the increase in vascular permeability (plasma leakage) that differentiates DHF from DF [6]. By the end of the febrile phase, DSS may occur, which is characterized by hypovolemic shock due to plasma leakage. Unusual manifestations (or expanded dengue syndrome) have been increasingly reported with involvement of severe organ impairment such as liver, kidneys, brain or heart. These may be associated with coinfections, comorbidities or complications of prolonged shock [6].

The DENV genome consists of a ~10.7 kb single-stranded positive-sense RNA genome encoding 3 structural (C, prM/M, E) and 7 non-structural (NS1, NS2A, NS2B, NS3, NS4A, NS4B, NS5) proteins [7]. DENV has very diverse genetic characteristics. The four antigenically-related serotypes differ by ~25–40% at the amino acid levels. Within each serotype, there are several clusters of variants termed as genotypes which vary by ~6% and 3% at the nucleotide and amino acid levels, respectively [8,9].

Dengue severity has been correlated with viral genetics. All four of the serotypes of DENV can cause severe and fatal disease, although DENV-2 and DENV-3 have been more associated with severe disease [10–13]. In Indonesia, all four of the DENV serotypes are circulating, with the tendency of DENV-3 related to severe diseases [14,15]. However, due to the limited serotype data available in Indonesia, it is possible that other serotypes also contribute to the severity of the disease.

Surabaya and Jakarta were the cities where dengue disease was first reported in Indonesia in 1968 [16]. Currently, all 34 provinces of Indonesia have reported dengue cases [14]. Dengue disease is quite common in urban areas in Indonesia, and it occurs annually, while periodic major outbreaks have occurred, such as those reported in 1998 [17] and 2004 [15]. In 2011, the East Java Provincial Health Office reported 1,008 dengue cases in Surabaya (incidence rate 36/100,000) with a case fatality rate of 0.70%. Although dengue in Surabaya has been reported [18,19], the clinical aspects of the disease and its correlation with virological factors have never been reported. Our study described the clinical features of dengue disease in Surabaya, combined with molecular analysis of DENV.

## Materials and methods

### Patient recruitment, sample collection and clinical and laboratory examinations

This cross-sectional study was performed from February to August 2012 in Surabaya, the capital city of East Java province, Indonesia. Surabaya is the second largest city in Indonesia; it covers an area of approximately 333,063 km^2^ and is inhabited by roughly 3 million people. Patients suspected of having dengue with fever >38°C accompanied by at least one of the symptoms of dengue such as headache, rash, arthralgia, retro-orbital pain, malaise, signs of DHF or DSS, presenting at the Dr. Soetomo Central Hospital were invited to participate in the study and were enrolled upon obtaining written consent. Consent for minors was obtained from parents or legal guardians. Ethical clearance for this study was obtained from Airlangga University Medical Research Ethics Committee. Sera from dengue-suspected patients were collected during the 3–5 days of fever and subjected to serology tests and dengue antigen detection. Anti-dengue IgG and IgM detections were performed using Panbio Dengue Duo IgM and IgG Capture ELISA (Alere, Brisbane, Australia), which was also used to determine the infection status (primary or secondary infection) according to manufacturer’s protocol. Briefly, a positive IgM result (> 11 of Panbio units) was indicative of active primary or secondary infection. An IgG-positive result (> 22 Panbio units) was indicative of active secondary infection. Primary infection was determined by positive IgM (> 11 Panbio units) and negative IgG (< 22 Panbio units), while secondary infection was determined by positive IgG (> 22 Panbio units), which could be accompanied by elevated IgM levels. Detection of DENV NS1 antigen detection was performed using a Panbio Dengue Early Rapid kit (Alere), according to the manufacturer’s instructions. All of the patients underwent examination 2–4 times of complete blood count, aspartate aminotransferase (AST), alanine transaminase (ALT), and albumin. Occurrences of hepatomegaly, splenomegaly, ascites, pleural effusion and perinephric fluid were examined using ultrasonographic methods. Classification of the clinical manifestations of dengue was based on the WHO SEARO 2011 dengue guideline [6] and we categorized patients <15 years as children [6].

### RNA extraction and reverse transcriptase-polymerase chain reaction (RT-PCR)

Virus RNA was extracted from serum samples using a MagNA Pure LC Total Nucleic Acid Isolation Kit and automated MagNA Pure LC 2.0 Instrument (Roche, Mannheim, Germany), according to manufacturer’s instructions. DENV nucleic acid detection and serotyping were performed using Simplexa^™^ Dengue Molecular Assay quantitative real-time RT-PCR [20] performed in a 3M Integrated Cycler machine (Focus Diagnostic, Cypress, CA, USA). Detailed methods for the Simplexa^™^ Dengue Molecular Assay were as described by the manufacturer.

### DENV genome copy number determination

Virus copy number examination was performed to quantify the numbers of DENV genome copy numbers in the sera of patients during the 3–5 days of fever. The quantitative real-time RT-PCR (qRT-PCR) was based on conventional two step PCR used for the detection of DENV [21]. Virus RNA was reverse-transcribed into cDNA and used in subsequent quantitative PCR steps with a Power SYBR Green PCR kit and an ABI 7500 machine (Applied Biosystems, Foster City, CA). A recombinant plasmid harboring DENV structural genes (C, prM/M, E) was generated using a Zero Blunt TOPO PCR Cloning kit (Invitrogen-Life Technologies, Carlsbad, CA, USA) and was serially diluted into known concentrations of the plasmid-cloned dengue genome and used as the genome copy number standard.

### Virus isolation using cell culture

The C6/36 cell line was used in virus isolation from RT-PCR-positive sera. A monolayer of cells was inoculated with 200 μl of sera in 2 ml of 1X RPMI medium supplemented with 2% of FBS, 2 mM of L-glutamine, 100 U/ml of penicillin, and 100 μg/ml of streptomycin (all from Gibco-Life Technologies, Carlsbad, CA, USA). Flasks were incubated for 1 hour at 28°C to allow for virus attachment. Following the incubation period, the inoculation medium was discarded and replenished with 3 ml of fresh medium. Infected cells were incubated at 28°C for 14 days.

### DENV genotyping

The genotyping of DENV was performed based on the Envelope (E) gene sequence. DENV RNAs were extracted from tissue-culture supernatant and were reverse-transcribed into cDNA using Superscript III reverse transcriptase (RT) (Invitrogen-Life Technologies) and DENV-specific primers. PCR amplifications were then performed using *Pfu* Turbo DNA Polymerase (Stratagene-Agilent Technologies, La Jolla, CA, USA). PCR products were purified from 0.8% agarose gel using a QIAquick gel extraction kit (Qiagen, Hilden, Germany) and were used in cycle sequencing reactions, performed using 6 overlapping primers from both strands and BigDye Dideoxy Terminator sequencing kits, version 3.1 (Applied Biosystems), according to the methods described by the manufacturer. DNA sequencing was performed on 3130xl genetic analyzer (Applied Biosystems) at the Eijkman Institute sequencing facility. The primers used in genotyping were described previously [22]. The resulting sequence reads were assembled using SeqScape, version 2.5 (Applied Biosystems), with additional manual adjustment performed when manual inspection of the assembly showed some discrepancies. The obtained E gene sequences have been deposited in GenBank (Table 5). Sequence alignment and initial dataset preparation were undertaken using MEGA software, version 5.0 [23]. Multiple sequence alignment was performed using MUSCLE [24] to generate sequence alignment representing the E protein segment. A dataset for each serotype was prepared using BEAUti, version 1.8.2, [25] followed by phylogenetic reconstruction and evolutionary rate analysis using Bayesian Markov chain Monte Carlo (MCMC) methods, as implemented in BEAST, version 1.8.2, [26] using a GTR+Γ_4_ model with invariant sites, a relaxed uncorrelated lognormal molecular clock and prior Bayesian skyline, with 100 million generations sampled for every 1,000^th^ iteration. MCMC traces were analyzed using Tracer, version 1.5.0, and optimization was applied to obtain an adequate effective sampling size (ESS) for all parameters. A maximum clade credibility (MCC) tree was created using TreeAnnotator, version 1.8.2, and was visualized in FigTree, version 1.4.0, which are available with the BEAST package. Genotyping was based on classifications by Goncalvez et al. [27], Twiddy et al. [28], Lanciotti et al. [29] and Lanciotti et al. [30] for DENV-1, -2. -3 and -4, respectively.

### Statistical analysis

Statistical analysis was performed using SPSS Statistics software, version 17.0 (SPSS Inc., Chicago, IL), and R statistical software (http://www.r-project.org). The significance of factors influencing disease severity were assessed using generalized logistic regression as implemented in *rms* library from R statistical software. A probability value of *p* < 0.05 was considered statistically significant.

## Results

### Patients’ characteristics and clinical manifestations

**Fig 1:**
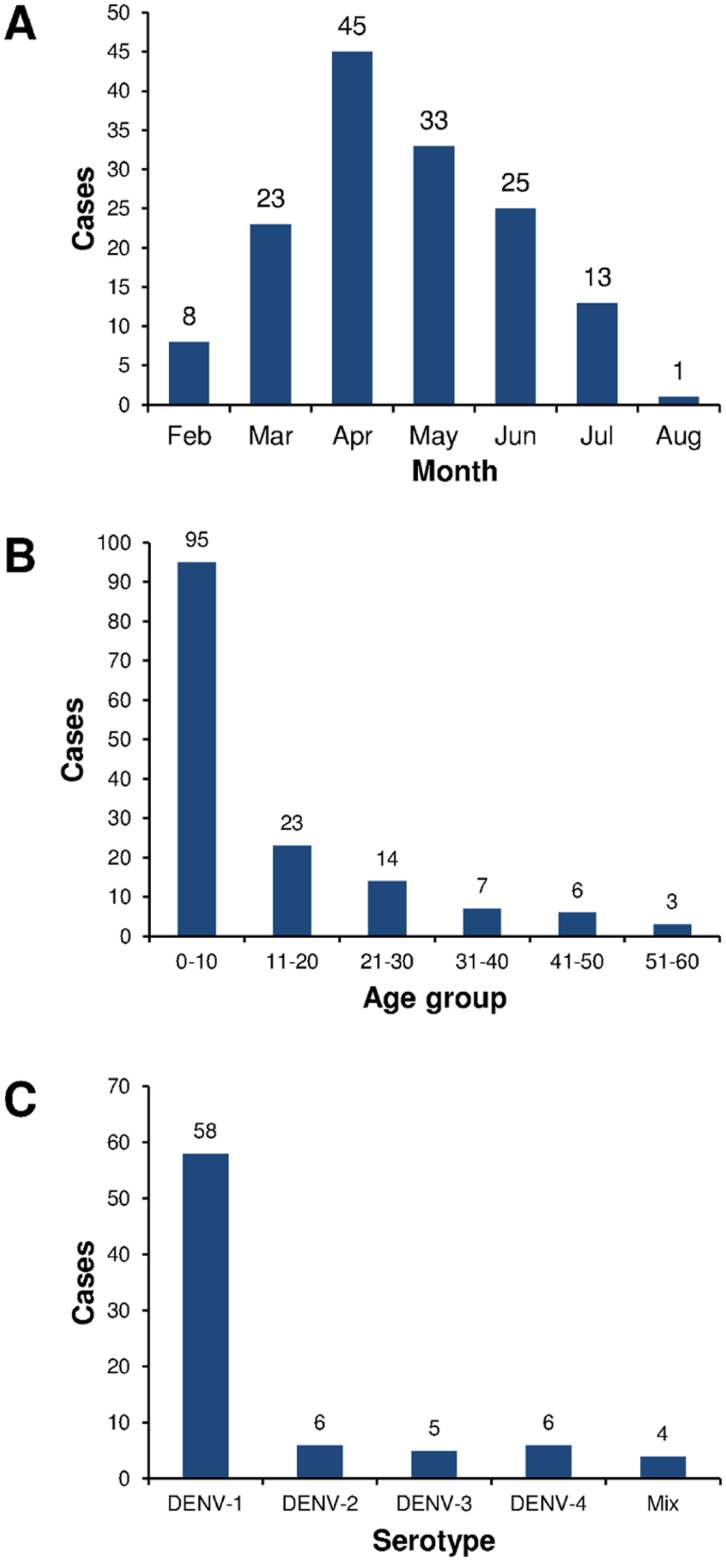
Dengue cases by monthly distribution (A), patients’ ages (B), and DENV serotype distribution (C) in Surabaya during 2012.

We recruited 148 dengue-suspected patients in this study after informed consent was obtained. From February to August 2012, the highest number of suspected dengue cases occurred in April and then decreased gradually (Fig 1A). Of the 148 patients, 101 (68%) of them were children (< 15 y.o.), and 47 (32%) were adults. Patients’ ages ranged from 0 to 60 y.o. Most of the dengue cases reported in this study occurred in children younger than 10 y.o. (Fig 1B). In terms of sex, 70 (47%) patients were male, and 78 (53%) patients were female (Table 1). The clinical manifestations of the patients, grouped according to the WHO-SEARO 2011 guideline [6], were as described in Table 1, in which most of the patients equally manifested as either DF or DHF (68 patients or 46% each). We also observed the presence of undifferentiated fever and expanded dengue syndrome among the patients (Table 1). The four patients with expanded dengue syndrome were all children with febrile seizures.

**Table 1:** Characteristics of dengue-suspected patients in Surabaya.

Characteristics (n = 148)	N	Percentage (%)
**Sex**		
Male	70	47
Female	78	53
Age grouping*		
Children	101	68
Adult	47	32
**Diagnosis**		
Undifferentiated Fever	8	5
Dengue Fever	68	46
Dengue Hemorrhagic Fever	68	46
Expanded Dengue Syndrome	4	3
**RT-PCR detection**		
Positive	79	53
Negative	69	47

*Age grouping based on children < 15 y.o.

### DENV serotype distribution in Surabaya

DENV molecular detection and serotyping were performed in all 148 collected sera. Of these sera, RT-PCR detection was positive in 79 samples (53%). Serotyping revealed the predominance of DENV-1, which accounted for 58 cases or 73%, followed by DENV-2 and DENV-4 (6 cases each or 8%) and then by DENV-3 (5 cases or 6%) (Fig 1C). The remainder of the confirmed dengue cases were detected as mixed infection of DENV-1 and -2 (1 case), DENV-1 and -3 (2 cases), and DENV-1 and -4 (1 case) (Fig 1C).

### Clinical features and laboratory examinations

Of the 79 dengue-confirmed patients, 67 patients had complete clinical and laboratory data. The age distribution of the patients was not equal between children and adults. The literature reported that patient age is one of the factors influencing the clinical presentation of dengue [31]. To analyze the clinical features and laboratory parameters, we grouped our patients into children (n = 48) and adults (n = 19). Significant differences in clinical/laboratory parameters were observed in the children. As expected, severity markers such as hematocrit, thrombocytes, liver enzymes and albumin were more prominent in DHF pediatric patients. Hematocrit was higher in DHF patients, as well as the AST and ALT enzymes. The platelet counts were significantly lower in the DHF group. Plasma leakage markers, such as gall bladder wall edema, ascites, and pleural effusion, were also observed in children with DHF (Table 2). Similarly, DHF occurred more as a secondary infection in children. In adult patients, less prominent markers of severity were observed. Only thrombocytopenia and pleural effusion were readily observed in DHF (Table 3). Unlike in children, viremia was significantly higher in adult DHF patients (Table 3). Other clinical/laboratory parameters were not significantly different in adult patients between DF and DHF (Table 3).

**Table 2:** Characteristics of dengue-confirmed children (n = 48).

Parameter	DF (n = 17)	DHF (n = 31)	*P*
Average Length of Stay (days)	3.7 ±1.3	4.2±0.8	0.449
Virus Titer (genome copy eq./μL)	262.5 ±314.9	2262.5±7781.9	0.207
Hemoglobin (g/dL)	**12.3±1.3**	**13.5±1.4**	0.008*
RBC count (x 10^6^/ μL)	**4.8±0.58**	**5.3±0.7**	0.016*
Hematocrit (%)	**37.7±3.6**	**40.5±4.1**	0.019*
MCV	78.4±4.8	76.3.1±6.3	0.249
MCH	25.8±1.7	25.6±2.2	0.923
MCHC	**32.8±1.1**	**32.6±5.5**	0.031*
WBC count (x 10^3^/ μL)	3.7±1.6	3.8±2.1	0.582
% Eosinophils	**1.7±1.9**	**0.8±1.4**	0.040*
% Basophils	1.2±1.2	1.5±1.5	0.589
% Neutrophils	32.0±16.4	50.9±78.7	0.232
% Lymphocytes	53.5±16.4	47.2±11.9	0.178
% Monocyte	10.8±4.1	13.1±5.1	0.106
Thrombocytes (x 10^3^/ μL)	**73.1±42.7**	**43.3±31.5**	0.003*
AST (IU/dL)	**92.2±39.8**	**206.31±194.6**	0.000*
ALT (IU/dL)	**37.2±29.3**	**74.8±70.3**	0.001*
Albumin (g/dL)	**3.3±0.4**	**2.7±0.6**	0.000*
Secondary infection	**9/17(52.9%)**	**26/31 (83.8%)**	0.039*
NS1 antigen positive	7/17(41.2%)	12/31(38.7%)	0.867
Hepatomegaly	2/17 (11.7%)	10/31(32.3%)	0.169
Gall bladder wall edema	**3/17 (17.6%)**	**23/31 (74.3%)**	0.000*
Splenomegaly	2/17 (11.7%)	1/31 (3.2%)	0.248
Perinephric fluid	0/17 (0%)	4/31 (12.9%)	0.282
Ascites	**0/17 (0%)**	**19/31 (61.3%)**	0.000*
Pleural Effusion	**0/17 (0%)**	**24/31 (77.4%)**	0.000*

MCH, mean corpuscular hemoglobin; MCV, mean corpuscular volume; MCHC, mean corpuscular hemoglobin concentration; AST, aspartate transaminase; ALT, alanine transaminase.

*Statistically significant

**Table 3:** Characteristics of dengue-confirmed adult patients (n = 19).

Parameter	DF (n = 9)	DHF (n = 10)	*p*
Average Length of Stay (days)	4.4 ±1.3	5.9±1.5	0.403
Virus Titer (genome copy eq./μL)	**202.5 ±164.1**	**324,770±9.08e5**	0.034*
Hemoglobin (g/dL)	13.2±1.8	14.5±164	0.008
RBC count (x 10^6^/μL)	4.6±0.6	5.0±0.7	0.165
Hematocrit (%)	39.2±5.1	42.3±5.1	0.142
MCV	86.1±5.9	84.7±3.9	0.249
MCH	28.4±1.3	28.5±1.1	0.153
MCHC	33.4±0.9	33.5±1.5	0.806
WBC count (x 10^3^/μL)	3.6±1.4	3.4±1.7	0.514
% Eosinophils	1.8±2.4	0.45±1.1	0.084
% Basophils	1.6±1.9	0.9±0.8	0.870
% Neutrophils	45.9±21.3	56.9±16.8	0.288
% Lymphocytes	37.2±16.9	31.6±14.8	0.514
% Monocytes	13.4±4.9	10.1±4.8	0.191
Thrombocytes (x 10^3^/μL)	**82.0±34.2**	**37.7±29.1**	0.013*
AST (IU/dL)	101.0±34.3	139.8±81.9	0.414
ALT (IU/dL)	87.1±39.7	75.8±60.8	0.327
Albumin (g/dL)	3.5±0.3	3.2±056	0.093
Secondary infection	8/9(88.8%)	8/10(80%)	1.000
NS1 antigen positive	6/9(66.6%)	6/10(60%)	1.000
Hepatomegaly	2/9 (22.2%)	2/10 (20%)	1.000
Gall bladder wall edema	1/9 (11.1%)	5/10 (50%)	0.141
Splenomegaly	0/9 (0%)	0/10 (0%)	N/A
Perinephric fluid	0/9 (0%)	1/10 (10%)	1.000
Ascites	0/9 (0%)	4/10 (40%)	0.303
Pleural Effusion	**0/9 (0%)**	**5/10 (50%)**	0.022*

*Statistically significant

### Correlations of DENV serotypes/genotypes with clinical manifestations and laboratory parameters

Different DENV serotypes have been reported to cause different clinical manifestations and disease severity. In regard to this fact, we sought to determine whether each DENV serotype was correlated with the clinical and laboratory data of the patients. In all of the patients with the infecting DENV serotypes determined, we did not observe any significant difference in clinical/laboratory data except for lymphocyte counts (Table 4). We observed a relatively higher lymphocyte number in patients infected by DENV-1, compared to other serotypes. The severity of the disease, which was grouped into DF and DHF, was not significantly different among serotypes. However, in all of the serotypes, the numbers of DHF cases were higher compared to DF cases (Table 4). Additionally, ANOVA test on logistic regression of disease severity with NS1 antigen detection, infection status, DENV serotype, age, and sex as cofactors indicated that the general influential factor in determining the disease severity was the infection status (*p* = 0.021, S1 Table).

**Table 4:** Clinical and laboratory parameters of patients grouped according to infecting serotypes.

Parameter	DENV-1 (n = 48)	DENV-2 (n = 6)	DENV-3 (n = 5)	DENV-4 (n = 6)	Mix (n = 2)	*p* value
*Severity (%)*						0.775^a^
DF	16 (33.3)	1 (16.7)	2 (40.0)	2 (33.3)	0 (0.0)	
DHF	32 (66.7)	5 (83.3)	3 (60.0)	4 (66.7)	2 (100)	
*Infection status (%)*						0.205^a^
Primary infection	16 (33.3)	1 (16.7)	0 (0.0)	0 (0.0)	1 (50.0)	
Secondary infection	32 (66.7)	5 (83.3)	5 (100)	6 (100)	1 (50.0)	
*Antigen detection (%)*						0.211^a^
NS1 antigen-positive	31 (64.6)	3 (50.0)	2 (40.0)	1 (16.7)	1 (50.0)	
NS1 antigen-negative	17 (35.4)	3 (50.0)	3 (60.0)	5 (83.3)	1 (50.0)	
*Viral load*^c^ *(mean ± SD)*	1502.2± 6221.9	205.7± 221.1^d^	189.8± 89.0	362.1± 238.3	N/A	0.909^b^
*Laboratory test (mean ± SD)*						
HB (g/dL)	12.90±1.5	11.62±1.0	12.48±0.9	13.07±1.1	10.80±2.4	0.090^b^
WBC (x10^3^/μL)	4.22±2.2	7.17±4.6	2.92±0.6	4.45±2.1	3.58±1.6	0.118^b^*
Lymphocyte (%)	49.19±16.6	36.24±21.7	33.30±20.5	36.00±6.9	26.65±15.9	**0.035**^b^
Hematocrit (%)	38.44±4.4	35.03±3.1	38.04±3.1	39.38±3.4	32.10±7.1	0.100^b^
Platelet (x10^3^/μL)	80.45±52.3	72.98±43.7	91.40±33.8	97.50±64.0	79.00±31.1	0.917^b^
AST (IU/dL)	135.05±115.0	107.67±58.6	111.40±61.6	80.33±81.9	568.50±733.3	0.137^b^*
ALT (IU/dL)	51.10±45.5	71.83±56.8	42.20±19.7	44.33±29.0	148.50±177.5	0.586^b^*
Albumin (g/dL)	3.30±0.5	3.13±0.8	3.42±0.5	3.57±0.6	3.25±0.4	0.745^b^
*USG examination (%)*						
Hepatomegaly	17 (35.4)	2 (33.3)	1 (20.0)	3 (50.0)	1 (50.0)	0.856^a^
Gall Bladder Wall Edema	22 (45.8)	3 (50.0)	3 (60.0)	1 (16.7)	0 (0.0)	0.448^a^
Splenomegaly	6 (12.5)	1 (16.7)	0 (0.0)	0 (0.0)	1 (50.0)	0.518^a^
Perinephric Fluid	3 (6.3)	1 (16.7)	0 (0.0)	0 (0.0)	0 (0.0)	0.786^a^
Ascites	15 (31.3)	3 (50.0)	2 (40.0)	0 (0.0)	1 (50.0)	0.524^a^
Pleural Effusion	21 (43.8)	3 (50.0)	3 (60.0)	3 (50.0)	0 (0.0)	0.757^a^

^a^ Pearson’s Chi-Square test

^b^ One-way ANOVA test

^c^ Viral genome copy number Equivalent/μL

^d^ One outlier was excluded from calculation

N/A: not applicable

* Statistical analysis was performed using Log_10_-transformed data to generate equal variances among groups.

We also analyzed the correlation between two genotypes of DENV-1 (described below) with clinical manifestations and laboratory parameters, however, no statistically significant correlation was found (data not shown).

### Phylogenetic analyses and DENV genotype distribution

**Fig 2:**
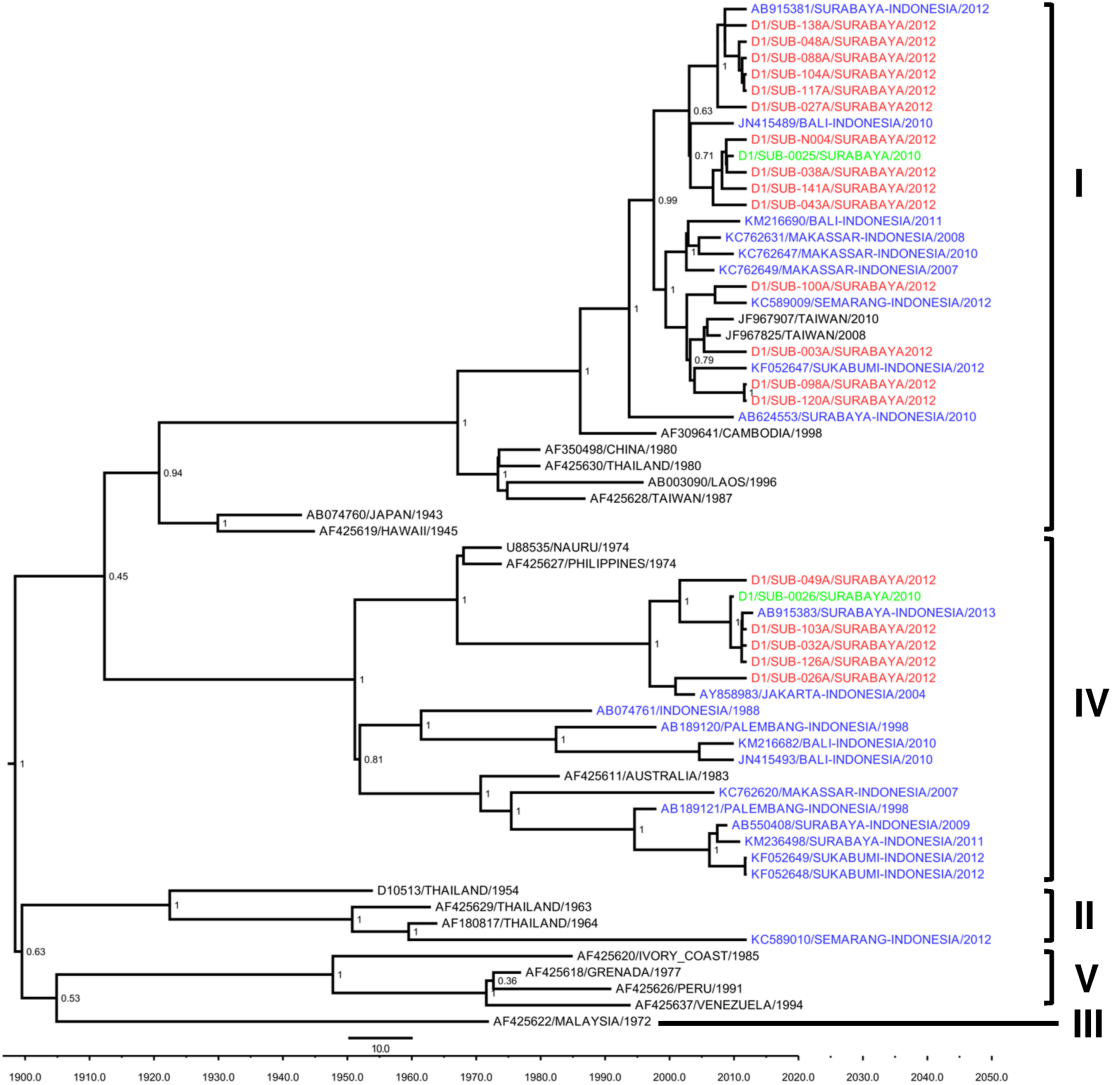
Maximum clade credibility (MCC) tree of DENV-1 genotype groupings generated by Bayesian inference method as implemented in BEAST using the GTR evolution model and gamma parameter rates from the E gene sequences. The Surabaya 2012 isolates (red font) were grouped into Genotype I and Genotype IV based on classification by Goncalvez et al [27], together with isolates from Surabaya 2010 (green font) and other cities in Indonesia (blue font). The posterior probabilities of the clades are indicated as numbers in the node labels.

To study the genetic diversity of the DENV, we performed genotyping of 24 DENV isolates, representing all of the serotypes, using the Envelope gene sequences for phylogenetic analysis. We also included five Surabaya DENV isolates collected in 2010 as references (Table 5). Of the 58 DENV-1 positive samples, we managed to sequence the Envelope genes of 19 virus isolates. Based on the DENV-1 genotype classification by Goncalvez et al [27], we observed the circulation of two genotypes of DENV-1 in Surabaya. The majority of isolates (14 isolates) were grouped into Genotype I, while the remainder (5 isolates) were grouped into Genotype IV (Fig 2).

**Table 5:** Surabaya DENV isolates with their Envelope genes sequenced.

No	Isolate ID	Serotype	Genotype	Clinical Manifestation	GenBank Accession No.
1.	SUB-003A	DENV-1	I	DHF	KT204436
2.	SUB-026A	DENV-1	IV	DHF	KT204437
3.	SUB-027A	DENV-1	I	DHF	KT204438
4.	SUB-032A	DENV-1	IV	DHF	KT204439
5.	SUB-048A	DENV-1	I	DHF	KT204440
6.	SUB-088A	DENV-1	I	DF	KT204441
7.	SUB-098A	DENV-1	I	DF	KT204442
8.	SUB-100A	DENV-1	I	DHF	KT204443
9.	SUB-103A	DENV-1	IV	DF	KT204444
10.	SUB-104A	DENV-1	I	DHF	KT204445
11.	SUB-120A	DENV-1	I	DF	KT204446
12.	SUB-126A	DENV-1	IV	DHF	KT204447
13.	SUB-138A	DENV-1	I	DF	KT204448
14.	SUB-N004	DENV-1	I	DF	KT204449
15.	SUB-117A	DENV-1	I	DHF	KT204450
16.	SUB-141A	DENV-1	I	DHF	KT204451
17.	SUB-038A	DENV-1	I	DF	KT204452
18.	SUB-049A	DENV-1	IV	DHF	KT204453
19.	SUB-043A	DENV-1	I	DHF	KT204454
20.	SUB-019A	DENV-2	Cosmopolitan	DHF	KT204455
21.	SUB-083A	DENV-3	I	DF	KT204456
22.	SUB-114A	DENV-3	I	DHF	KT204457
23.	SUB-124A	DENV-3	I	DF	KT204458
24.	SUB-022A	DENV-4	II	DHF	KT204459
25.	SUB-0025	DENV-1	I	N/A	KT204460
26.	SUB-0026	DENV-1	IV	N/A	KT204461
27.	SUB-0027	DENV-3	I	N/A	KT204462
28.	SUB-0030	DENV-3	I	N/A	KT204463
29.	SUB-0007	DENV-4	II	N/A	KT204464

Note: N/A: data not available; the last 5 isolates were collected in 2010

**Fig 3:**
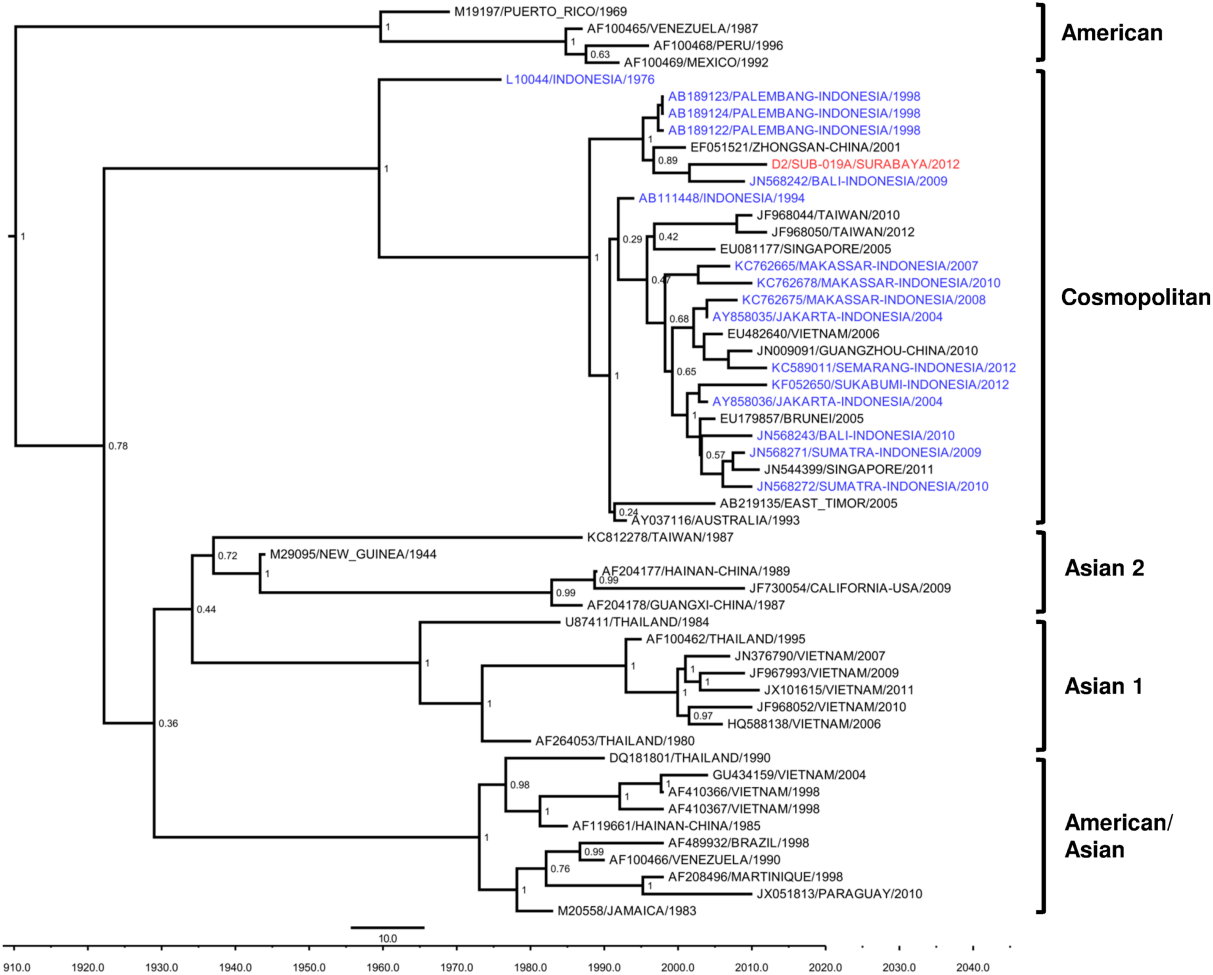
MCC tree of DENV-2 genotype groupings generated by Bayesian inference method as implemented in BEAST using the GTR evolution model and gamma parameter rates from the E gene sequences. The Surabaya 2012 isolate (red font) was grouped into Cosmopolitan Genotypes based on classification by Twiddy et al [28], together with isolates from other cities in Indonesia (blue font). The posterior probabilities of the clades are indicated as numbers in the node labels.

For DENV-2, the isolate was classified as the Cosmopolitan genotype (Fig 3), according to Twiddy et al’s [28] classification. Using this classification tree, the isolate was grouped together with DENV-2 isolates from other cities in Indonesia (Bali and Palembang). Further analysis of the Cosmopolitan genotype of DENV-2, using sets of sequences from Indonesia from recent years, revealed that the Surabaya 2012 isolate was grouped into the Surabaya lineage of the Cosmopolitan subclade, as proposed by Kotaki, et al. [32] (data not shown). The isolate is closely related to an isolate of imported DENV from Indonesia in Taiwan in 2007 [33], and it shares common ancestors with isolates from Bali, Singapore, and Surabaya in 2011.

**Fig 4:**
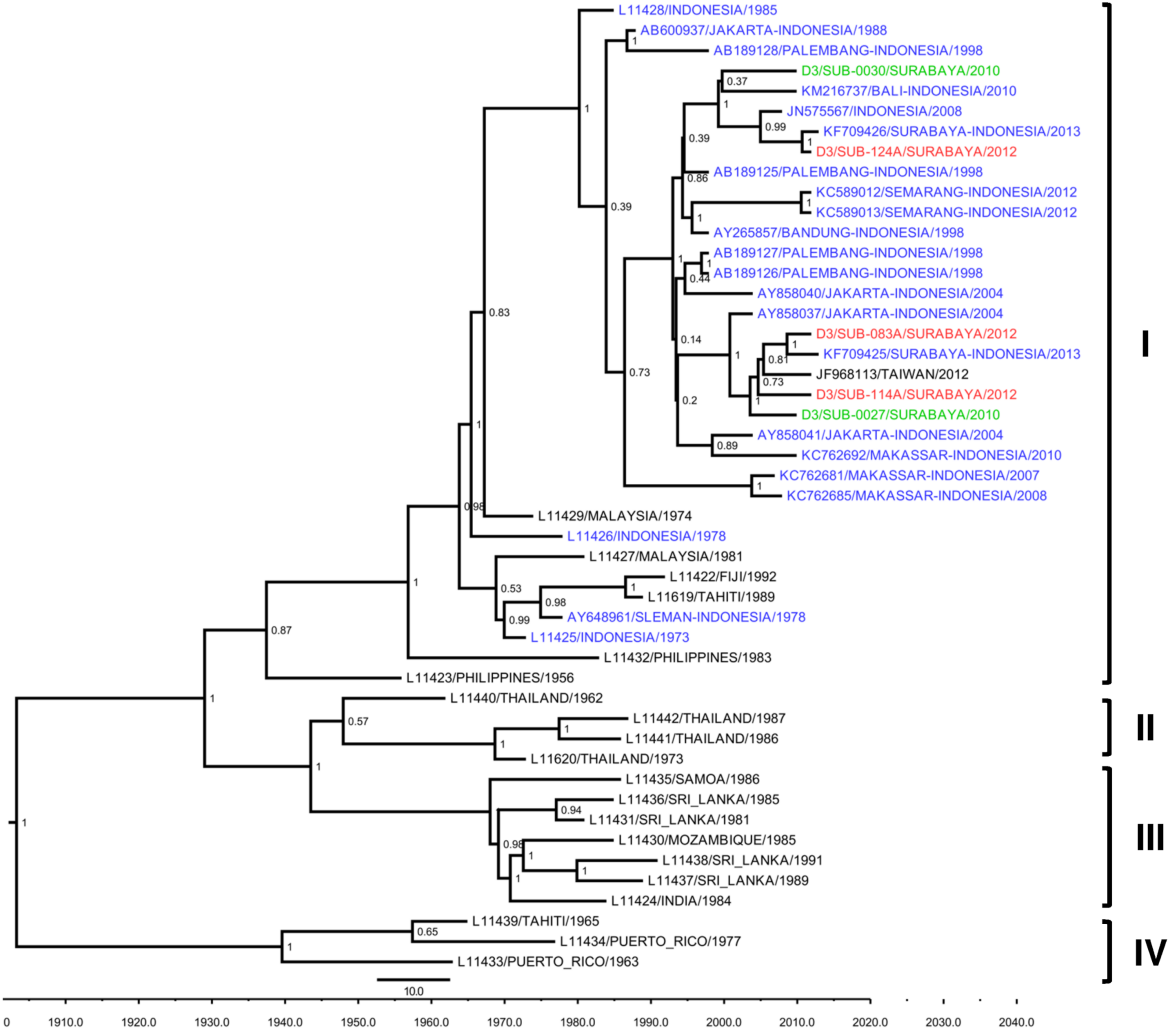
MCC tree of DENV-3 genotype groupings generated by Bayesian inference method as implemented in BEAST using GTR evolution model and gamma parameter rates from the E gene sequences. The Surabaya 2012 isolates (red font) were grouped into Genotype I based on classification by Lanciotti et al [29], together with isolates from Surabaya 2010 (green font) and other cities in Indonesia (blue font). The posterior probabilities of the clades are indicated as numbers in the node labels.

The genotypes of DENV-3 isolates were classified as Genotype I according to Lanciotti et al [29]. These DENV-3 isolates apparently formed two separate clusters within Genotype I. However, in each cluster, the 2012 isolates grouped together with other isolates from Surabaya and other location in Indonesia, such as Jakarta (2004) and Bali (2010), as well as the recent Surabaya 2013 isolates. The Taiwan isolate from an imported case in Indonesia also clustered together with the Surabaya 2012 isolates (Fig 4).

**Fig 5:**
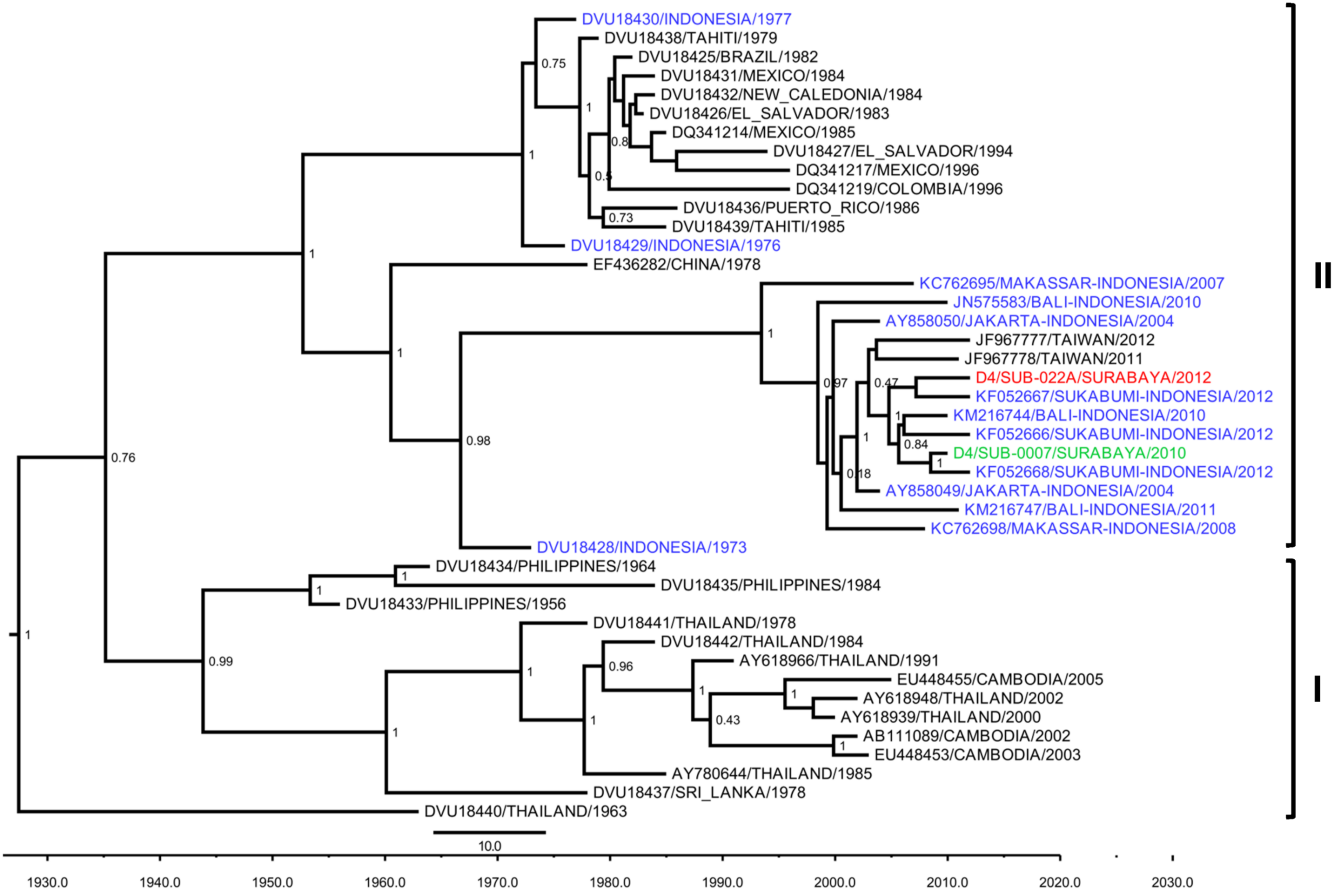
MCC tree of DENV-4 genotype groupings generated by Bayesian inference method as implemented in BEAST using GTR evolution model and gamma parameter rates from the E gene sequences. The Surabaya 2012 isolate (red font) was grouped into Genotype II based on classification by Lanciotti et al [30], together with isolates from Surabaya 2010 (green font) and other cities in Indonesia (blue font). The posterior probabilities of the clades are indicated as numbers in the node labels.

We managed to sequence one DENV-4 isolate and performed phylogenetic analysis based on Lanciotti et al’s classification [34] to determine the genotype. As shown in Fig 5, the isolate from Surabaya was classified as Genotype II, and it clustered together with isolates from other locations in Indonesia, such as from Sukabumi [35], Bali [36], and Makassar [22].

### Envelope gene amino acid analysis

**Fig 6:**
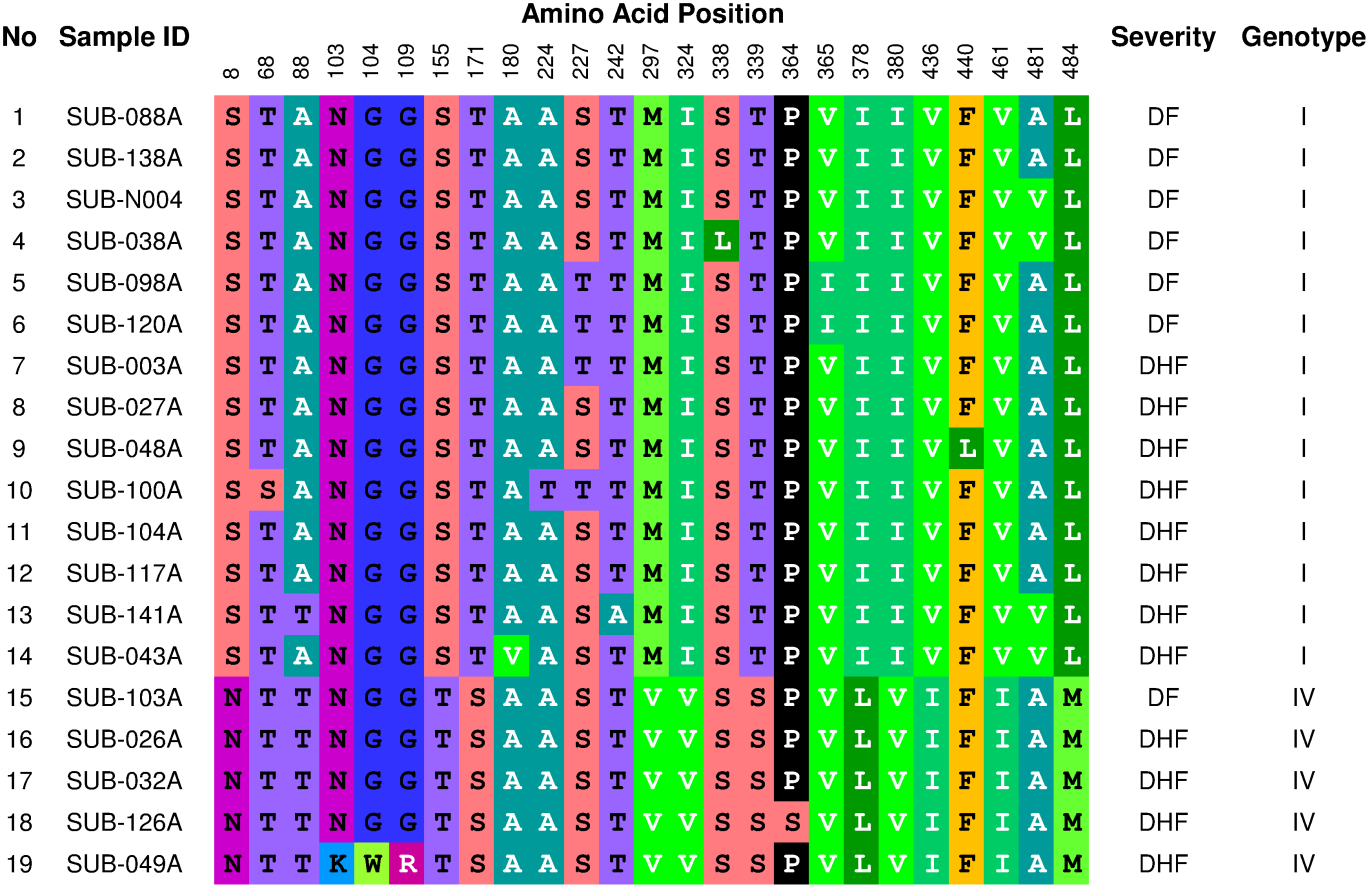
Comparative analysis of amino acid substitutions within the Envelope protein among Surabaya DENV-1 viruses. Only variable amino acids are shown.

With the available E gene DNA sequences obtained in this study, we analyzed the amino acid (AA) sequences of the E glycoprotein of 19 DENV-1 isolates to determine whether there is an AA substitution uniquely related to the disease severity. As shown in Fig 6, there were 25 of 495 (5%) AAs that were variable within the 19 isolates. Notably, there was a clear difference in AA sequences between Genotype I and Genotype IV isolates (Fig 6). The substitutions of AAs were mostly conservative, such as threonine to serine and isoleucine to valine substitutions (Fig 6). The AA substitutions apparently randomly occurred in isolates associated with both DF and DSS. We did not observe any unique AA substitution(s) related to disease severity. Because only a small number isolates were sequenced for DENV-2, -3, and -4, we did not perform AA comparisons.

## Discussion

We reported here the clinical observations and virological features of dengue in Surabaya. During the study, we recruited 148 dengue-suspected patients. In Surabaya, dengue cases occurred throughout the months of February through August 2012, with cases peaking in April (Fig 1A). High dengue incidence in April-May is typically observed in Indonesia, especially in large cities such as Jakarta, Surabaya, and Bandung [37].

The majority of patients (68%) were children younger than 15 years old. This finding was also similar to dengue cases described earlier in Indonesia, i.e., in Jayapura in 1993 [38], Palembang in 1998 [17], and Semarang in 2012 [39], but different from what we reported previously in Sukabumi in 2012 [35] and Makassar in 2007–2010 [22], in which most of the cases occurred in adolescent and adult patients. The fact that more children patients observed in Surabaya was not align with the tendency of dengue incidence shifting from young children to older age groups in Indonesia [40].

In our study, within all of the age groups, we observed more female than male dengue patients (Table 1). However, in adult patients, more dengue incidents in men were observed. These data are in accordance with reports from six countries in Asia that consistently observed the predominance of male dengue patients [41]. Although more study is needed to confirm the cause, it is possible that, in Surabaya, adult men have greater exposure to dengue-carrying mosquitoes at workplaces or while travelling to and from work.

Our DENV serotyping of Surabaya samples in 2012 revealed the circulation of all four dengue serotypes, with DENV-1 predominantly circulating in the region, while quite similar numbers for DENV-2; -3; and -4 were observed (Fig 1C). This result was different from previous reports, which described only DENV-1 and DENV-2 being found in Surabaya in 2012 [18]. Previous dengue outbreaks in Indonesia have been attributed primarily to DENV-3 [17,38,42,43], but our recent studies indicated that DENV-1 has become the predominant serotype in outbreaks in several cities [22,39]. Our serotype data also showed the exchange of DENV serotype predominance in Surabaya, i.e., from DENV-2 in 2008–2009 [44] to DENV-1 in 2012. Overall, our data on the predominance of DENV-1 in Surabaya in 2012 and continued in 2013, as reported previously [18], suggested that the DENV-1 has become the predominant serotype in Surabaya within the last three to four years since 2009. Other serotypes, i.e., DENV-2, -3 and -4, were continuously circulating, albeit at lower numbers.

Regarding the clinical aspects of dengue in Surabaya, we observed the equal occurrence of DF and DHF in our patients. Our findings showed that disease severity, as manifested by DF and DHF, was not related to specific serotype. Previous reports from Indonesia observed that all serotypes could cause severe dengue [45]. Similarly, our previous data also did not find any direct correlation between the infecting serotypes and disease severity [39], as did another report [46]. A recent report described DENV-1 as more related to severe disease and more likely presenting with red eyes [47]. In our study, we did not specifically examine red eyes as a clinical sign; therefore, we do not know whether, in our study, DENV-1 was also related to red eyes. A limitation of our data was that the serotype distribution was not equal in our patients, with the DENV-1 being predominant, which might cause result bias.

In our study, we grouped our patients into children and adult patients. It has been reported that the patient’s age is one of the factors influencing dengue clinical presentation [31]. As observed in Tables 2 and 3, more prominent signs of clinical manifestations and hematology findings commonly found in DHF, such as the hemoconcentration, thrombocytopenia, elevated liver enzymes and albumin, [6] were observed in children with DHF. Furthermore, evidence of plasma leakage, as indicated by the occurrence of pleural effusion, gall bladder wall edema, and ascites, [6] was more observed in DHF than DF (Table 2). In adult patients, although most of the clinical signs and hematology findings were consistent with the WHO classification for DF and DHF, only viral load, thrombocytopenia, and pleural effusion were statistically significant (Table 3). Our findings were consistent with a previous study that reported that the frequency of symptoms and signs in the WHO classification schemes was reduced significantly with increasing age of infection [48].

Related to dengue confirmation using NS1 antigen detection, we observed relatively low sensitivity of NS1 in both children and adult (39.5% and 63.1%, respectively) in RT-PCR positive samples (Tables 2 and 3). These low numbers were in accordance with previous studies describing the low sensitivity of NS1 detection in Indonesia [49,50].

Changes in lymphocyte subsets in dengue fever have been recognized previously [51,52]. In our study, compared to other serotypes, both lymphocyte counts and viral load were highest in DENV-1 (Table 4). Although correlation between viral load and serotypes was not statistically significant, we found that correlation between lymphocyte count and serotypes was significant (*p* = 0.035). Other studies described that viral load and/or lymphocyte count were associated with the infecting serotypes [47,53]. As such, the relationship between DENV serotypes, viremia level, and lymphocyte count warrants further studies.

Studies have revealed that a higher viral load is a risk factor for severe disease, in which patients with DHF had higher viral loads than patients with DF [54,55]. Our study revealed similar findings, in which viral load were higher in DHF compared to DF (Table 3). The occurrence of more DHF in patients with secondary infections was also observed in our study (Table 2), and further regression analysis indicated that infection status affected the severity of the diseases (S1 Table). This is consistent with the observation that secondary infection is one of the risk factors for severe dengue [56].

We observed a similar co-circulation of DENV-1 Genotypes I and IV with the previous reports in Surabaya in 2012 [18,19], as well as our DENV-1 isolates collected in 2010 (Fig 2 and Table 5). Based on the number of isolates, Genotype I apparently started to predominate over Genotype IV, a condition similar to the DENV-1 genotype distribution in Makassar [22]. Examining further detail, we observed the grouping of DENV-1 Genotype I in Surabaya into two major clades (Fig 2). The upper clade, in which most of the Surabaya isolates were grouped, contains isolates from the nearby city of Denpasar, Bali [36], while the lower clade contains isolates from Bali [36], Semarang [39], Makassar [22], and Sukabumi [35]. The Taiwan isolates, which originated from Indonesia as imported cases, [57] were also grouped in this clade (Fig 2). We do not know whether the different clade presence in Surabaya was correlated with the viral fitness. The grouping of Surabaya isolates with DENV from other cities in Indonesia suggested that the circulating DENV-1 viruses are local and endemic strains.

The DENV-2 Surabaya isolate was grouped closely with isolates from Bali and Palembang and, together with isolates from other areas in Indonesia such as Jakarta, Semarang, Sukabumi, Makassar and Sumatra, was classified into the Cosmopolitan genotype (Fig 3). This genotype is quite commonly found in Southeast Asia, including Indonesia [28]. Further analysis of the Cosmopolitan genotype, using Indonesia DENV isolates from recent years, revealed that the Surabaya 2012 isolate was grouped together into a subclade proposed as the Surabaya lineage, which grouped the majority of isolates from Surabaya during the period of 2008–2014 [32]. The Surabaya 2012 isolate shares common ancestors with the DENV isolate from Taiwan, which was imported from Indonesia. The shared common ancestor of Indonesia DENV-2 with Taiwan isolates imported from Indonesia has also been reported in Semarang, Central Java [39]. The data of DENV-2 from this study contributed to the addition of DENV-2 genetic information from 2012 and suggested the endemic nature of the DENV-2 in Surabaya over the years.

Phylogenetic analysis of three Surabaya DENV-3 isolates grouped them into Genotype I (Fig 4), which is a common genotype found in Indonesia, such as in isolates from Jakarta, Palembang, Semarang, Makassar, Sukabumi, Bandung, Sleman, and Bali [17,22,35,36,39,58]. Thus, the DENV-3 circulating in Surabaya most likely consisted of local, endemic strains that have been circulating for decades in Indonesia. For DENV-4, the phylogenetic analysis determined this isolate as Genotype II (Fig 5). This genotype is also commonly found in Indonesia, as depicted by the grouping of isolates from many cities in Indonesia into Genotype II (Fig 5). Overall, we observed that the DENVs circulating and infecting people in Surabaya were from local, endemic strains that dynamically circulate in the city.

The DENV genotypes are known to differ in both fitness and virulence [8]. Certain genotypes of DENV have been accounted for as being risk factors for severe disease [59–61]. For example, the lineage replacement of American DENV-2 by Asian/American DENV-2 has been well documented in Puerto Rico [62]. The DENV-3 Genotype IV never been associated with DHF, while Genotype III were frequently associated with DHF outbreaks [29]. DENV-3 Genotype II was associated with severe epidemics in Nicaragua, Guatemala, and Mexico [63–65]. In another example, a distinct subgroup of DENV-3 Genotype III appeared at the same time with the emergence of DHF in Sri Lanka in 1989 [60]. In Nicaragua, an abrupt increase of disease severity was observed during DENV-2 transmission which coincided with replacement of Asian/American DENV-2 NI-1 clade with a new virus clade, NI-2B [66]. Although a large body of evidence has accumulated for the correlation between DENV genotypes and disease severity, in our study we detected no statistically significant correlations. In Surabaya, the DENV-1 Genotype I and IV were co-circulating. Both genotypes were capable to cause both DF and DHF. Analysis of clinical and hematological findings also did not observe any significant correlation with DENV-1 genotypes (data not shown). In addition, specific amino acids and nucleotide substitutions responsible for viral virulence have been studied [9,67]. Likely the most studied amino acid mutation is D390N in the E protein, which affects viral replication [67,68]. As such, we compared the genetics of DENV-1 Genotypes I and IV using the E gene AA sequences (Fig 6). We revealed clear differences in AA sequence variations between these two genotypes. However, the AA comparison did not reveal any specific mutations related to disease severity. The AA substitutions were shared by isolates causing both DF and DHF (Fig 6). Previous studies have also observed similar findings and have found no reproducible genetic differences related to disease severity [69–72]. However, we are aware that only E protein AA sequences were compared in this study. It is possible that other virulence determinants are present within the dengue genome, such as in the 5’ and 3’ untranslated regions (UTRs), which have been associated with disease severity [67,68]. Altogether, our study did not find any direct relationship between DENV serotypes/genotypes and disease severity. We are aware that our analyses may be limited by the relatively small sample size. Therefore, future study using larger sample size will be beneficial in determining the relationships between viral genetics and disease severity. In addition, studies comparing the whole genomes of DF- and DHF-associated isolates will be useful for finding the genetic determinants of viral virulence.

In summary, we revealed the clinical and virological aspects of dengue in Surabaya. DF and DHF equally occurred in our patients. Between children and adult patients, the clinical manifestations and symptoms of dengue in Surabaya were more prominent in children. All of the DENV serotypes circulated, with the DENV-1 as the predominant serotype. No associations of serotypes/genotypes with disease severity were observed.

## Supporting information

S1 Table ANOVA result based on logistic regression of the disease severity on various factors. (PDF)
