# Supplementary material for: Clinical and virological characteristics of dengue in Surabaya, Indonesia
Source: PLoS One. 2017 Jun 2;12(6):e0178443. doi: 10.1371/journal.pone.0178443 (PMC5456069; doi:10.1371/journal.pone.0178443)
Supplement: S1 Table — (PDF) [file pone.0178443.s001.pdf]

S1 Table. ANOVA result based on logistic regression of the disease severity on various factors.

Factor	χ^2	df	*p* value
**NS1 antigen detection**	0.55	1	0.460
**Infection status**	5.29	1	**0.021**
**DENV Serotype**	1.45	4	0.835
**Age**	1.32	1	0.251
**Sex**	0.71	1	0.401

df: degree of freedom

χ^2: Chi-square statistics, *p*-value < 0.05 is considered statistically significant (printed in bold)

Logistic regression model: **Severity ~ NS1 antigen detection + Infection status + DENV Serotype + Age + Sex**
